# Carbolic Acid Poisoning

**Published:** 1881-10

**Authors:** 


### CARBOLIC ACID POISONING.

The patient (service of Dr. Sands, N. Y., Hospital) had been subjected to the operation of amputation of the thigh. For five days after the urine was green in color, due to absorption, by the wound of carbolic acid used in accordance with the antiseptic method of operating.—*The Med. Record, N. Y.*

